# Comparison of Clinical Characteristics and Short-Term Prognoses Within Hospitalized Chronic Obstructive Pulmonary Disease Patients Comorbid With Asthma, Bronchiectasis, and Their Overlaps: Findings From the ACURE Registry

**DOI:** 10.3389/fmed.2022.817048

**Published:** 2022-02-25

**Authors:** Jieping Lei, Ting Yang, Chen Liang, Ke Huang, Sinan Wu, Chen Wang

**Affiliations:** ^1^Data and Project Management Unit, Institute of Clinical Medical Sciences, China-Japan Friendship Hospital, Beijing, China; ^2^Department of Pulmonary and Critical Care Medicine, Center of Respiratory Medicine, China-Japan Friendship Hospital, Beijing, China; ^3^Institute of Respiratory Medicine, Chinese Academy of Medical Sciences, Beijing, China; ^4^National Clinical Research Center for Respiratory Disease, Beijing, China; ^5^National Center for Respiratory Medicine, Beijing, China; ^6^Chinese Alliance for Respiratory Diseases in Primary Care, Beijing, China; ^7^Chinese Academy of Medical Sciences, Peking Union Medical College, Beijing, China; ^8^Department of Respiratory Medicine, Capital Medical University, Beijing, China

**Keywords:** chronic obstructive pulmonary disease, exacerbation, asthma, bronchiectasis, phenotype, heterogeneity, prognosis

## Abstract

**Introduction:**

Real-world evidence and comparison among commonly seen chronic obstructive pulmonary disease (COPD) phenotypes, i.e., asthma–COPD overlap (ACO), bronchiectasis–COPD overlap (BCO), and their coexistence (ABCO) have not been fully depicted, especially in Chinese patients.

**Methods:**

Data were retrieved from an ongoing nationwide registry in hospitalized patients due to acute exacerbation of COPD in China (ACURE).

**Results:**

Of the eligible 4,813 patients with COPD, 338 (7.02%), 492 (10.22%), and 63 (1.31%) were identified as ACO, BCO, and ABCO phenotypes, respectively. Relatively, the ABCO phenotype had a younger age with a median of 62.99 years [interquartile range (IQR): 55.93–69.48] and the COPD phenotype had an older age with a median of 70.15 years (IQR: 64.37–76.82). The BCO and COPD phenotypes were similar in body mass index with a median of 21.79 kg/m^2^ (IQR: 19.47–23.97) and 21.79 kg/m^2^ (IQR: 19.49–24.22), respectively. The COPD phenotype had more male gender (79.90%) and smokers (71.12%) with a longer history of smoking (median: 32.45 years, IQR: 0.00–43.91). The ACO and ABCO phenotypes suffered more prior allergic episodes with a proportion of 18.05 and 19.05%, respectively. The ACO phenotype exhibited a higher level of eosinophil and better lung reversibility. Moreover, the four phenotypes showed no significant difference neither in all-cause mortality, intensive care unit admission, length of hospital stay, and COPD Assessment Test score change during the index hospitalization, and nor in the day 30 outcomes, i.e., all-cause mortality, recurrence of exacerbation, all-cause, and exacerbation-related readmission.

**Conclusions:**

The ACO, BCO, ABCO, and COPD phenotypes exhibited distinct clinical features but had no varied short-term prognoses. Further validation in a larger sample is warranted.

## Introduction

Patients with chronic obstructive pulmonary disease (COPD), comorbid with asthma (asthma–COPD overlap, ACO) or bronchiectasis (bronchiectasis–COPD overlap, BCO) as well as their coexistence (ABCO), are commonly seen phenotypes, which have been broadly discussed whether they were distinct disease entities, but there is no concluded consensus yet (1). The concept of “ACO” or “asthma + COPD” appeals as a great interest to investigators (2–11), and similar discussion applies to the research of bronchiectasis and COPD overlap (12–16). Few data was reported on the ABCO phenotype.

The prevalence of the above-mentioned phenotypes of COPD varied across studies. Alshabanat A et al. reported a pooled prevalence of ACO phenotype among patients with COPD was 27% and 28% in population- and hospital-based studies, respectively. Hosseini et al. (17) found that a pooled prevalence of ACO phenotype was 29.6% in patients with COPD. Zhou et al. (18) stated an ACO prevalence of 11.51% in Chinese patients with COPD. Ding et al. (19) reported an ACO prevalence of 18.6% in urban Chinese patients with COPD. Uchida et al. (20) summarized the prevalence of ACO phenotype that varied from 0.9 to 11.1% in the general population, from 11.1 to 61.0% in patients with asthma, and from 4.2 to 66.0% in patients with COPD (21). For the BCO phenotype, Ni et al. (22) reported a pooled prevalence of 54.3% (ranging from 25.6 to 69%) in patients with COPD.

Both asthma and bronchiectasis could incur an exacerbation of the chronic pulmonary disease (AECOPD). The ACO and BCO phenotypes present common and distinct clinical characteristics and prognoses. In general, the ACO phenotype exhibits a higher eosinophil level and better bronchodilator reversibility, and the BCO phenotype presents more neutrophil and nonreversible characteristics (23, 24). Additionally, compared with patients with COPD only, the ACO phenotype was characterized with higher probability of exacerbation (5), frequent outpatient and emergency department visits (25, 26), but lower rate of hospital readmission (27) and mortality (28, 29). The BCO phenotype was associated with increased risk of exacerbation, severe airway obstruction, and higher mortality (30).

Although numerous studies have been conducted, findings were still controversial (1). The notable variation may be due to different study designs, sample sizes, and definitions of diseases used. In addition, most previous data were based on stable stage of COPD, and facts on exacerbation of the disease need to be delineated. In the current manuscript, to address these unanswered questions, we utilized data from an acute exacerbation of COPD inpatient registry (ACURE) to investigate the differences in clinical features and short-term prognosis profiles among Chinese hospitalized patients with AECOPD who were comorbid with asthma and/or bronchiectasis, and those patients without the two comorbidities. We anticipated that our findings could help improve clinical management of the disease in clinical practice.

## Methods

### Study Design and Settings

Data was retrieved from an acute exacerbation of COPD inpatient registry (ACURE), which was initiated in China to investigate the demographic characteristics, clinical features, diagnoses and treatments, and prognoses among hospitalized patients with COPD who were suffering an acute exacerbation episode (ClinicalTrials.gov registry number: NCT02657525). The ACURE study was started on September 1, 2017 and planned to recruit 7,600 hospitalized patients with AECOPD who were admitted to 161 participating medical centers across China with a maximum of 3-year follow-up. The protocol of the ACURE registry and baseline characteristics of the study population have been published (31, 32).

The study protocol, informed consent, and case report form have been approved by the institutional review board at the China–Japan Friendship Hospital (approval number: 2015-88) and other local participating centers. All the participating patients have provided written informed consent.

### Study Population

The ACURE participants underwent screenings at index hospital admission to confirm their eligibilities for enrollments. Subjects would be enrolled if they fulfilled the following eligibility criteria: 1) aged 18 years or older; 2) confirmed or suspected to be hospitalized due to AECOPD; 3) not participating in other clinical trial or intervention studies; and 4) agreeing to sign the informed consent. As of February 25, 2020, 4,813 eligible patients met the inclusion and exclusion criteria.

### Procedures and Measurements

#### Data Collection

In the ACURE registry, patient management was at the discretion of clinical physicians. Well-designed and sophisticated questionnaires were administrated to enrolled participants at baseline, i.e., during the index hospitalization, and at planned follow-up visits, i.e., at day 30 (±2 days), month 6 (±12 days), month 12 (±12 days), month 24 (±12 days), and month 36 (±12 days), respectively after the index hospital discharge by trained investigators. The questionnaires consisted of contents on basic and demographic information, inclusion and exclusion criteria, current diagnoses (including symptoms and signs), objective examinations [e.g., routine venous blood, lung function, computed tomography (CT), arterial blood gas, electrocardiograph, cardiac color ultrasound, pulmonary ventilation/perfusion image, lower extremity venous ultrasound, and etiological examinations where necessary], history and management of the disease (especially while in the stable condition), predisposing factors and prevention of the exacerbation, pharmacological and nonpharmacological (e.g., respiratory support) treatments in the hospital, cost, outcomes at hospital discharge, and management and outcomes during the follow-ups. Data of any exacerbations that did not occur in the scheduled visits were also collected.

All data were uploaded to an online electronic data capture system. Data quality was regularly monitored by a concerted project and data management team. For instance, missing values, outliers, and illogical information will be sent to the local participating centers for timely amendment.

#### Diagnoses of Diseases

In current analyses, spirometric COPD was diagnosed as the presence of a post-bronchodilator forced expiratory volume in 1 s (FEV1) divided by the forced vital capacity (FVC) with a value of less than 0.70, which indicated a persistent airflow limitation according to the Global Initiative for Chronic Obstructive Lung Disease (GOLD) 2021 report (10). Asthma was diagnosed by the presence of both variable expiratory airflow limitation and a characteristic pattern of respiratory symptoms for instance wheezing, shortness of breath (dyspnea), chest tightness, and/or cough in adults according to the Global Initiative for Asthma (GINA) 2021 report (9). Bronchiectasis was diagnosed by the presence of both bronchial dilation on CT and clinical symptoms, such as cough, sputum production, and/or recurrent respiratory infection in adults according to the European Respiratory Society (33) and British Thoracic Society guidelines (34). The ACO phenotype was defined as spirometry-diagnosed COPD and asthma. The BCO phenotype was confirmed by spirometric COPD and CT-based bronchiectasis. Patients with COPD both comorbid with asthma and bronchiectasis were termed ABCO phenotype. Information on disease diagnoses were obtained from patients' medical records during their index hospitalization and critically reviewed by local and central principal investigators/pulmonary physicians. More details on diagnoses of diseases are provided in the Supplementary Material S1.

#### Variables

Data on demographic and clinical characteristics, laboratory, lung function, and image (e.g., CT) tests, short- and long-term prognoses were comprehensively collected. Lung function test was performed when the situation of the patient was relatively stable after admission. If multiple lung function tests were conducted, the latest result was chosen before discharge. Bronchiectasis was diagnosed once any of the CT scan result was confirmed during hospitalization. Classification of the severity of airflow limitation was categorized into four grades based on the 2021 GOLD report: GOLD 1 (mild, FEV1 ≥ 80% predicted), GOLD 2 (moderate, 50% ≤ FEV1 < 80% predicted), GOLD 3 (severe, 30% ≤ FEV1 < 50% predicted), and GOLD 4 (very severe, FEV1 < 30% predicted) (10).

In this manuscript, short-term clinical outcomes referred to the length of index hospital stay, recurrence of exacerbation, and exacerbation-related hospital readmission within 30 days after the index hospital discharge. Definitions of clinical outcomes are explicated in the Supplementary Material S1.

### Statistical Analyses

Mean and standard deviation (SD) were calculated for normally distributed continuous variables, otherwise median and interquartile range (IQR) were presented for abnormally distributed ones. Frequencies and percentages were calculated for categorical variables. Characteristics of patients within phenotypes were compared with the utilization of Student's *t*-test (normal distributed data, two-group comparison) or Wilcoxon rank-sum test (abnormal distributed data, two-group comparison) or Kruskal–Wallis test (abnormal distributed data, three or above group comparison) as appropriate for continuous variables, and using Pearson's Chi-square test or Fisher's exact test as appropriate for categorical ones.

A multivariable linear regression model was used to assess the associations of predictors with a continuous outcome (i.e., length of hospital stay). Multivariable Cox proportional hazards regression model was adapted to investigate the associations with day 30 outcomes (i.e., recurrence of exacerbation and exacerbation-related hospital readmission). Variables that showed significant associations in univariable analyses (*p* < 0.10), and factors (e.g., age, gender, BMI, smoking status, and FEV1) that previously had been reported to be associated with prognoses of COPD were further adjusted in the multivariable statistical models (35). A stepwise selection scheme with an entry level of 0.10 and a stay level of 0.05 was applied. Multicollinearity diagnosis was performed before conducting the multivariable analyses. Details on multivariable models employed for stepwise selection of independent predictors are given in the Supplementary Material S2. Kaplan–Meier curve and log-rank test were utilized to determine the differences in day 30 outcomes between phenotypes.

Statistical significance was defined as achieving a two-sided *P* value of less than 0.05. Statistical analyses were performed using Statistical Analysis System (version 9.4) and R Project (version 4.0.5).

## Results

**Figure 1:**
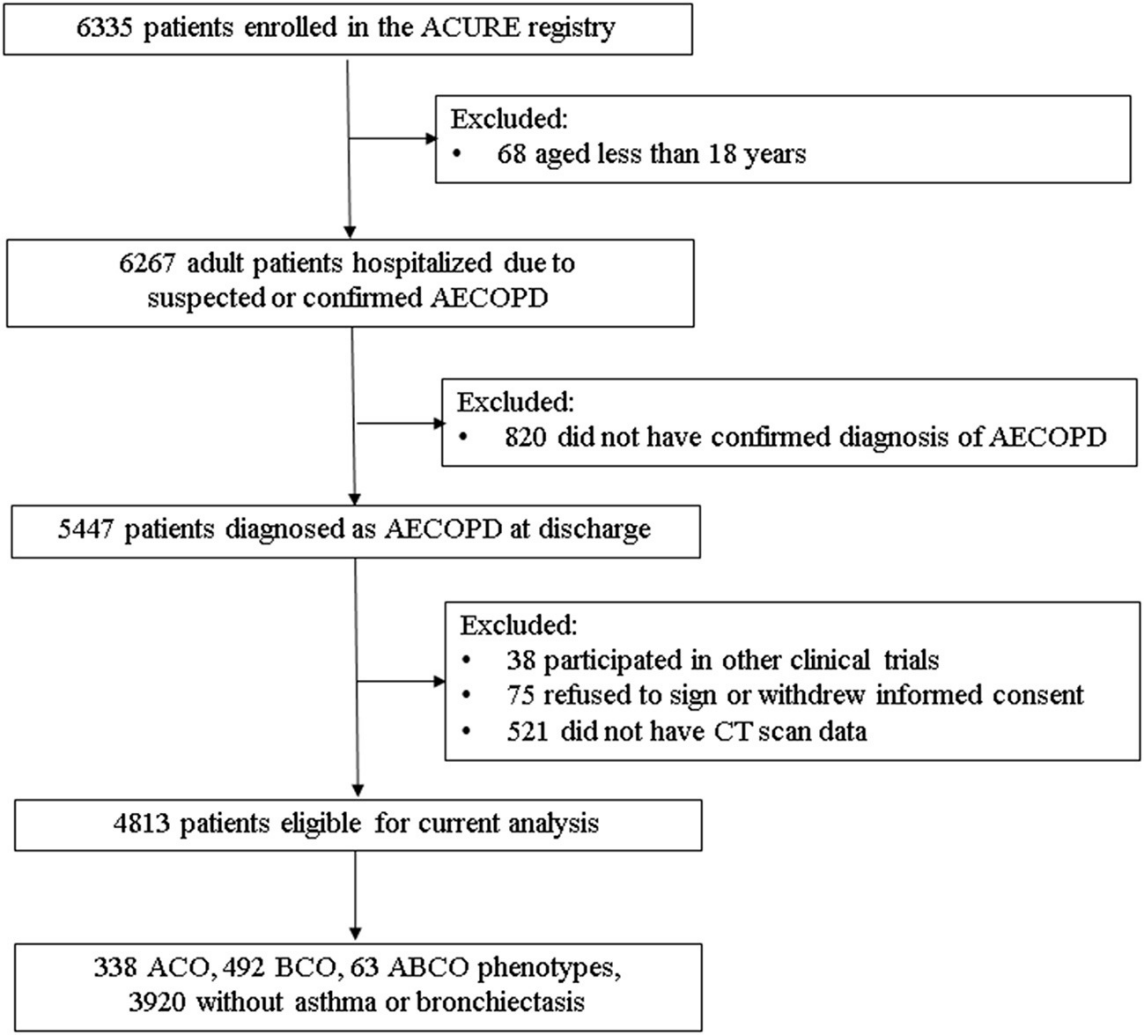
Flowchart of diagram in current analyses. ABCO, chronic obstructive pulmonary disease patients comorbid with asthma and bronchiectasis; ACO, asthma-chronic obstructive pulmonary disease overlap; ACURE, the acute exacerbation of chronic obstructive pulmonary disease inpatient registry; AECOPD, acute exacerbation of chronic obstructive pulmonary disease; BCO, bronchiectasis- chronic obstructive pulmonary disease overlap; CT, computed tomography.

Of the overall 4,813 eligible patients with AECOPD, 63 (1.31%) patients were comorbid both with asthma and bronchiectasis (ABCO), 338 (7.02%) and 492 (10.22%) patients were identified as ACO and BCO phenotypes, respectively, and 3,920 (81.45%) patients did not coexist with asthma or bronchiectasis (Figure 1).

Demographic and clinical characteristics among the ACO, BCO, and ABCO phenotypes as well as those patients without asthma or bronchiectasis are shown in Table 1. Relatively, the ABCO phenotype patients had a younger age with a median of 62.99 years (IQR: 55.93–69.48), and the patients without asthma or bronchiectasis had an older age with a median of 70.15 years (IQR: 64.37–76.82). The BCO phenotype patients and patients without asthma or bronchiectasis were similar in BMI with a median of 21.79 kg/m^2^ (IQR: 19.47–23.97) and 21.79 kg/m^2^ (IQR: 19.49–24.22), respectively. In addition, patients without asthma or bronchiectasis were more of the male gender (79.90%) and smokers (71.12%) with a longer history of smoking (median: 32.45 years, IQR: 0.00–43.91). The ACO and ABCO phenotype patients suffered more prior allergic episodes with a proportion of 18.05 and 19.05%, respectively. The ACO phenotype patients exhibited higher level of eosinophil and better lung reversibility. The four phenotype patients showed no significant difference in all-cause mortality, intensive care unit (ICU)/respiratory intensive care unit (RICU) admission, length of stay, and COPD assessment test (CAT) score change in the index hospitalization, and neither in the day 30 outcomes, i.e., all-cause mortality, recurrence of exacerbation, and all-cause and exacerbation-related hospital readmission (Table 1).

**Table 1:** Main characteristics and comparisons among ACO, BCO, ABCO phenotypes, and those without asthma or bronchiectasis in hospitalized AECOPD patients.

**Main variables**	**All patients (*N* = 4,813)**	**ACO phenotype (*N* = 338)**	**BCO phenotype (*N* = 492)**	**ABCO phenotype (*N* = 63)**	**Patients without asthma or bronchiectasis (*N* = 3,920)**	***P* value for ACO vs. BCO**	***P* value among four phenotypes**
**Demographics**							
Age (years), median (IQR)	69.70 (63.72, 76.46)	66.19 (59.63, 73.53)	69.14 (63.16, 75.44)	62.99 (55.93, 69.48)	70.15 (64.37, 76.82)	<0.0001	<0.0001
*Gender, n (%)*							
Male	3,751 (77.93)	232 (68.64)	342 (69.51)	45 (71.43)	3,132 (79.90)	0.7890	<0.0001
Female	1,062 (22.07)	106 (31.36)	150 (30.49)	18 (28.57)	788 (20.10)		
BMI (kg/m^2^), median (IQR)	21.97 (19.53, 24.24)	23.41 (20.57, 25.71)	21.79 (19.47, 23.97)	23.18 (20.20, 26.04)	21.79 (19.49, 24.22)	<0.0001	<0.0001
*Education level, n (%)*							
Primary school or below	2,372 (49.28)	155 (45.86)	238 (48.37)	23 (36.51)	1,956 (49.90)	0.0457	0.0766
Junior high school	1,570 (32.62)	107 (31.66)	177 (35.98)	23 (36.51)	1,263 (32.22)		
Senior high school	676 (14.05)	57 (16.86)	64 (13.01)	14 (22.22)	541 (13.80)		
Undergraduate or above	195 (4.05)	19 (5.62)	13 (2.64)	3 (4.76)	160 (4.08)		
*Smoking status, n (%)*							
Former	2,117 (43.99)	114 (33.73)	181 (36.79)	25 (39.68)	1,797 (45.84)	0.0124	<0.0001
Current	1,165 (24.21)	83 (24.56)	80 (16.26)	11 (17.46)	991 (25.28)		
Never	1,531 (31.81)	141 (41.72)	231 (46.95)	27 (42.86)	1,132 (28.88)		
Smoking years (years), median (IQR)	30.71 (0.00, 42.48)	22.50 (0.00, 40.00)	10.00 (0.00, 40.00)	20.00 (0.00, 33.00)	32.45 (0.00, 43.91)	0.2317	<0.0001
**Medical history**							
Previous allergic episode, n (%)	555 (11.53)	61 (18.05)	55 (11.18)	12 (19.05)	427 (10.89)	0.0050	0.0003
Vaccination within past 5 years, n (%)	137 (2.85)	11 (3.25)	10 (2.03)	2 (3.17)	114 (2.91)	0.2707	0.6914
Emergency visit due to AECOPD in prior year (times), median (IQR)	0.00 (0.00, 1.00)	0.00 (0.00, 1.00)	0.00 (0.00, 2.00)	0.00 (0.00, 1.00)	0.00 (0.00, 1.00)	0.4031	0.4868
Hospitalization due to AECOPD in prior year (times), median (IQR)	1.00 (0.00, 2.00)	0.00 (0.00, 1.00)	1.00 (0.00, 2.00)	1.00 (0.00, 2.00)	1.00 (0.00, 2.00)	0.0062	0.0279
**Other comorbidities, n (%)**							
*Respiratory disease*							
Community-acquired pneumonia	1,483 (30.81)	103 (30.47)	152 (30.89)	19 (30.16)	1,209 (30.84)	0.8972	0.9984
VTE (including PE and DVT)	43 (0.89)	5 (1.48)	2 (0.41)	0 (0.00)	36 (0.92)	0.1277	0.4201
Pulmonary interstitial fibrosis	121 (2.51)	10 (2.96)	11 (2.24)	2 (3.17)	98 (2.50)	0.5147	0.9093
Pulmonary heart disease	1,015 (21.09)	52 (15.38)	102 (20.73)	14 (22.22)	847 (21.61)	0.0515	0.0621
Failure of respiration	1,209 (25.12)	73 (21.60)	131 (26.63)	24 (38.10)	981 (25.03)	0.0983	0.0371
*Cardiovascular disease*							
Coronary heart disease	861 (17.89)	59 (17.46)	91 (18.50)	6 (9.52)	705 (17.98)	0.7020	0.3629
Acute heart failure	21 (0.44)	0 (0.00)	5 (1.02)	0 (0.00)	16 (0.41)	0.0836	0.1674
Chronic heart failure	277 (5.76)	22 (6.51)	25 (5.08)	3 (4.76)	227 (5.79)	0.3820	0.8279
*Digestive disease*							
Gastroesophageal reflux disease	91 (1.89)	12 (3.55)	12 (2.44)	3 (4.76)	64 (1.63)	0.3479	0.0184
Peptic ulcer	69 (1.43)	3 (0.89)	6 (1.22)	2 (3.17)	58 (1.48)	0.7450	0.4442
*Other condition*							
Cancer	55 (1.14)	4 (1.18)	5 (1.02)	1 (1.59)	45 (1.15)	1.0000	0.8488
Cerebrovascular disease	230 (4.78)	22 (6.51)	22 (4.47)	2 (3.17)	184 (4.69)	0.1981	0.4328
**Peripheral biomarkers, median (IQR)**							
White blood cell (*10^9^/L)	7.18 (5.57, 9.40)	7.42 (5.70, 9.38)	7.48 (5.76, 10.10)	7.71 (6.13, 10.27)	7.11 (5.51, 9.30)	0.3642	0.0123
Platelet (*10^9^/L)	0.02 (0.02, 0.03)	0.02 (0.02, 0.03)	0.02 (0.02, 0.03)	0.02 (0.02, 0.02)	0.02 (0.02, 0.03)	0.6041	<0.0001
Neutrophil (%)	67.90 (55.50, 77.60)	64.10 (53.30, 73.70)	70.00 (58.45, 79.10)	67.10 (57.30, 77.50)	67.90 (55.45, 77.67)	<0.0001	0.0006
Neutrophil (*10^9^/L)	5.02 (3.54, 7.20)	4.73 (3.30, 6.93)	5.34 (3.77, 7.86)	5.72 (4.60, 7.22)	4.97 (3.50, 7.19)	0.0027	0.0040
Lymphocyte (%)	16.22 (7.90, 24.40)	18.40 (7.80, 26.60)	15.10 (8.40, 23.20)	16.70 (7.20, 24.20)	16.21 (7.80, 24.30)	0.0124	0.0903
Lymphocyte (*10^9^/L)	1.27 (0.86, 1.75)	1.40 (0.94, 1.94)	1.23 (0.87, 1.70)	1.54 (1.10, 2.19)	1.26 (0.85, 1.73)	0.0097	0.0031
Eosinophil (%)	1.10 (0.10, 2.70)	1.40 (0.10, 4.10)	0.90 (0.10, 2.60)	0.90 (0.02, 2.30)	1.10 (0.10, 2.70)	0.0204	0.0407
Eosinophil (*10^9^/L)	0.10 (0.02, 0.21)	0.12 (0.03, 0.33)	0.09 (0.02, 0.20)	0.11 (0.04, 0.27)	0.10 (0.02, 0.21)	0.0266	0.1148
Direct bilirubin (umol/L)	3.20 (1.90, 4.70)	3.10 (1.90, 4.40)	3.10 (1.87, 4.60)	3.20 (2.20, 4.10)	3.20 (1.90, 4.70)	0.6448	0.6359
Alkaline phosphatase (U/L)	0.01 (0.01, 0.01)	0.01 (0.01, 0.02)	0.01 (0.01, 0.01)	0.01 (0.01, 0.01)	0.01 (0.01, 0.01)	0.1520	0.2242
Gamma glutathione transpeptidase (U/L)	0.00 (0.00, 8.80)	0.00 (0.00, 14.66)	0.00 (0.00, 0.02)	0.00 (0.00, 15.30)	0.00 (0.00, 7.80)	0.0644	0.1028
**Arterial blood gas, median (IQR)**							
PaCO_2_ (mmHg)	34.30 (0.00, 43.40)	33.10 (0.00, 41.20)	32.60 (0.00, 43.60)	0.00 (0.00, 40.90)	34.70 (0.00, 43.50)	0.0190	0.0038
PaO_2_ (mmHg)	0.00 (0.00, 68.90)	0.00 (0.00, 64.40)	0.00 (0.00, 65.25)	0.00 (0.00, 0.00)	0.00 (0.00, 69.90)	0.5230	0.0004
SaO_2_ (%)	0.00 (0.00, 94.80)	0.00 (0.00, 94.80)	0.00 (0.00, 93.80)	0.00 (0.00, 84.40)	68.30 (0.00, 94.90)	0.1940	0.0004
**Lung function test**							
*Post-bronchodilator lung function, median (IQR)*							
FEV1 (L)	0.93 (0.65, 1.32)	1.20 (0.87, 1.57)	0.87 (0.62, 1.17)	1.00 (0.74, 1.32)	0.91 (0.64, 1.31)	<0.0001	<0.0001
FVC (L)	1.97 (1.45, 2.54)	2.35 (1.77, 2.94)	1.79 (1.33, 2.27)	2.21 (1.73, 2.81)	1.95 (1.43, 2.53)	<0.0001	<0.0001
FEV1/FVC (%)	0.50 (0.42, 0.59)	0.54 (0.44, 0.61)	0.51 (0.43, 0.58)	0.44 (0.40, 0.52)	0.50 (0.42, 0.59)	0.0383	0.0003
FEV1 % predicted value (%)	0.40 (0.28, 0.56)	0.50 (0.36, 0.64)	0.38 (0.28, 0.51)	0.39 (0.33, 0.51)	0.39 (0.27, 0.56)	<0.0001	<0.0001
FVC % predicted value (%)	0.65 (0.50, 0.82)	0.77 (0.61, 0.90)	0.62 (0.48, 0.76)	0.67 (0.59, 0.79)	0.65 (0.49, 0.82)	<0.0001	<0.0001
*GOLD categories, n (%)*							
GOLD 1	246 (5.11)	23 (6.80)	22 (4.47)	1 (1.59)	200 (5.10)	<0.0001	<0.0001
GOLD 2	1,102 (22.90)	121 (35.80)	90 (18.29)	12 (19.05)	879 (22.42)		
GOLD 3	1,490 (30.96)	102 (30.18)	178 (36.18)	26 (41.27)	1,184 (30.20)		
GOLD 4	1,185 (24.62)	44 (13.02)	121 (24.59)	12 (19.05)	1,008 (25.71)		
**Short-term clinical outcomes**							
*Within the index hospitalization*							
All-cause mortality, n (%)	3 (0.06)	0 (0.00)	1 (0.20)	0 (0.00)	2 (0.05)	1.0000	0.4598
ICU/RICU admission, n (%)	70 (1.45)	6 (1.78)	4 (0.81)	0 (0.00)	60 (1.53)	0.3315	0.5494
Length of hospital stay (days), median (IQR)	10.00 (8.00, 13.00)	10.00 (8.00, 13.00)	10.00 (8.00, 13.00)	10.00 (8.00, 12.00)	10.00 (8.00, 13.00)	0.7748	0.8653
CAT score change between 4 weeks prior admission and discharge, median (IQR)	−7 (−12, −3)	−7 (−12, −3)	−7 (−12, −3)	−8 (−13, −3)	−7 (−12, −3)	0.8580	0.8526
*During 30 days after the index hospital discharge*							
All-cause mortality, n (%)	9 (0.19)	0 (0.00)	2 (0.41)	1 (1.59)	6 (0.15)	0.5166	0.0670
Recurrence of AECOPD, n (%)	205 (4.26)	11 (3.25)	25 (5.08)	4 (6.35)	165 (4.21)	0.2043	0.5028
All-cause readmission, n (%)	129 (2.68)	7 (2.07)	15 (3.05)	2 (3.17)	105 (2.68)	0.3889	0.8504
AECOPD-related readmission, n (%)	99 (2.06)	5 (1.48)	13 (2.64)	1 (1.59)	80 (2.04)	0.2584	0.6891

*ABCO, chronic obstructive pulmonary disease patients comorbid with asthma and bronchiectasis; ACO, asthma-chronic obstructive pulmonary disease overlap; AECOPD, acute exacerbation of chronic obstructive pulmonary disease; BCO, bronchiectasis- chronic obstructive pulmonary disease overlap; BMI, body mass index; CAT, chronic obstructive pulmonary disease assessment test; COPD, chronic obstructive pulmonary disease; DVT, deep venous thrombosis; FEV1, forced expiratory volume in 1 s; FVC, forced vital capacity; GOLD, the Global Initiative for Chronic Obstructive Lung Disease; ICU, intensive care unit; IQR, interquartile range; PE, pulmonary embolism; RICU, respiratory intensive care unit; SD, standard deviation*.

**Figure 2:**
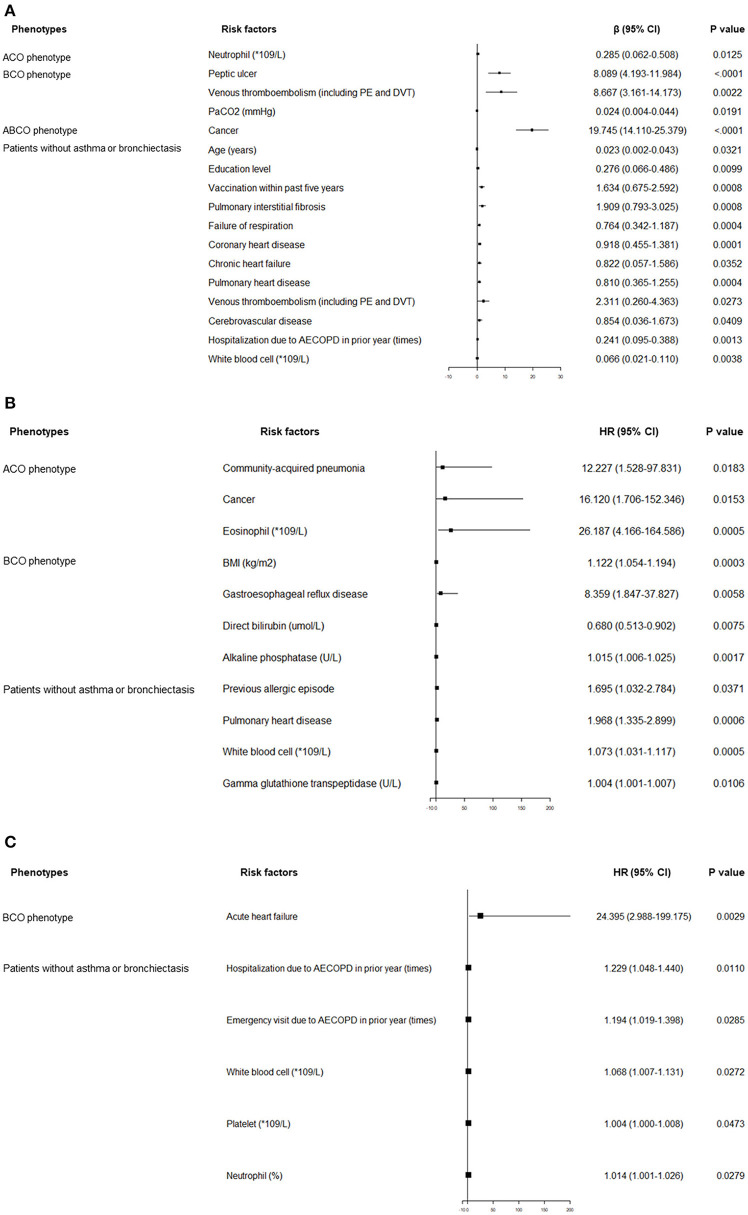
**(A)** Independent risk factors associated with length of index hospital stay. **(B)** Independent risk factors associated with recurrence of AECOPD within 30 days after the index hospital discharge. **(C)** Independent risk factors associated with exacerbation-related hospital readmission within 30 days after the index hospital discharge. ABCO, chronic obstructive pulmonary disease patients comorbid with asthma and bronchiectasis; ACO, asthma-chronic obstructive pulmonary disease overlap; AECOPD, acute exacerbation of chronic obstructive pulmonary disease; BCO, bronchiectasis-chronic obstructive pulmonary disease overlap; BMI, body mass index; CI, confidence interval; DVT, deep venous thrombosis; HR, hazard ratio; ICU, intensive care unit; PE, pulmonary embolism; RICU, respiratory intensive care unit.

Independent predictors associated with hospitalized and day-30 outcomes across the four phenotypes are shown in Figure 2. Varied factors were found in predicting the length of hospital stay among different phenotypes. For example, cancer was solely the strong predictor within the ABCO phenotype patients. Peptic ulcer, venous thromboembolism (VTE), and PaCO_2_ were the independent influencing factors in the BCO phenotype patients. More predicting factors such as age, education level, vaccination within the past 5 years, hospitalization due to AECOPD in the prior year, level of white blood cell, VTE, pulmonary interstitial fibrosis, failure of respiration, etc. were included in the profiles in the patients without asthma or bronchiectasis (Figure 2).

The predicting profiles of day 30 outcomes also exhibited distinct features. Community-acquired pneumonia, cancer, and the level of eosinophils were independent predictors of exacerbation recurrence in ACO phenotype patients. In BCO phenotype patients, BMI, gastroesophageal reflux disease, and level of alkaline phosphatase were associated with increased probabilities of exacerbation recurrence. Previous allergic episode, pulmonary heart disease, levels of white blood cells, and gamma glutathione transpeptidase were independent predictors in the patients without asthma or bronchiectasis. With respect to the exacerbation-related readmission, acute heart failure was the single strong predictor in the BCO phenotype patients. Times of hospitalization and emergency visit due to exacerbation in the prior year, levels of white blood cells and platelets, and percentage of neutrophils were positively associated with elevated exacerbation-related readmission rate in patients without asthma or bronchiectasis (Figure 2).

**Figure 3:**
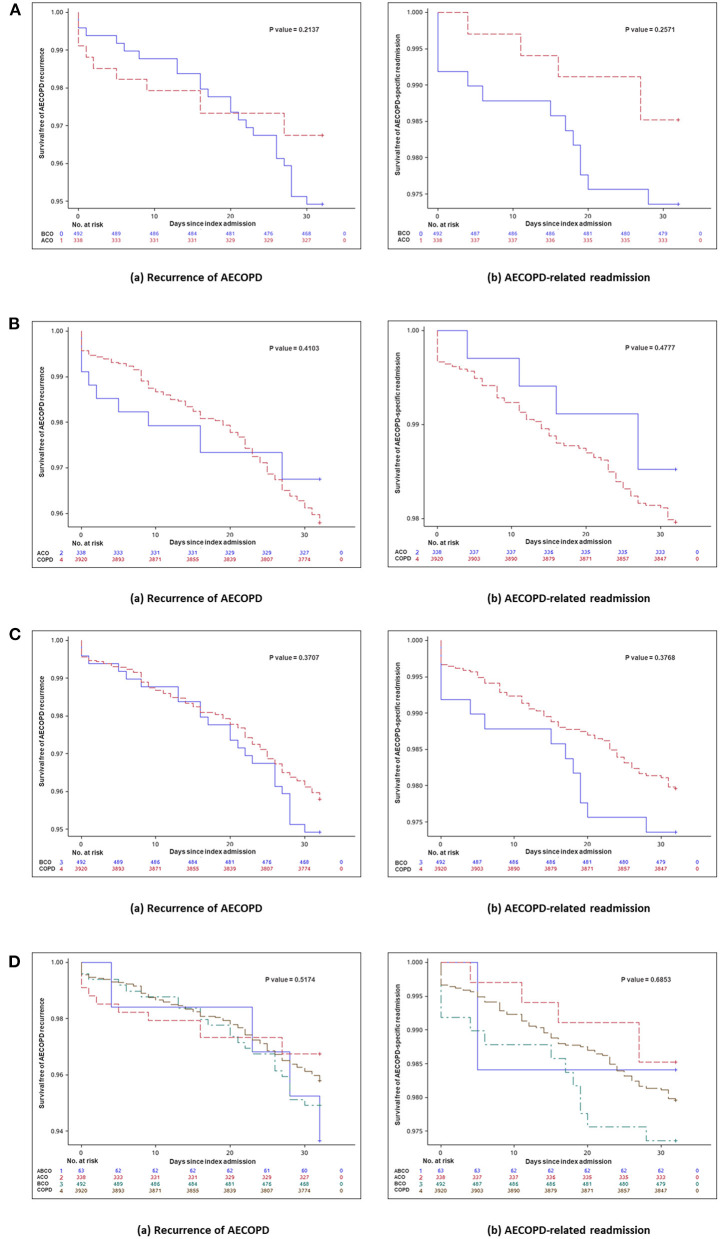
**(A)** Kaplan–Meier curve and log-rank test for day 30 outcomes between ACO and BCO phenotypes in hospitalized patients with AECOPD. Kaplan–Meier curve and log-rank test for day 30 outcomes (a) recurrent exacerbation (*P* = 0.2137) and (b) exacerbation-related readmission (*p* = 0.2571) between ACO and BCO phenotypes in hospitalized patients with AECOPD. **(B)** Kaplan–Meier curve and log-rank test for day 30 outcomes between ACO phenotype and patients without asthma or bronchiectasis in hospitalized patients with AECOPD. Kaplan–Meier curve and log-rank test for day 30 outcomes (a) recurrent exacerbation (*p* = 0.4103) and (b) exacerbation-related readmission (*p* = 0.4777) between ACO phenotype and patients without asthma or bronchiectasis in hospitalized patients with AECOPD. **(C)** Kaplan–Meier curve and log-rank test for day 30 outcomes between BCO phenotype and patients without asthma or bronchiectasis in hospitalized patients with AECOPD. Kaplan–Meier curve and log-rank test for day 30 outcomes (a) recurrent exacerbation (*P* = 0.3707) and (b) exacerbation-related readmission (*P* = 0.3768) between BCO phenotype and patients without asthma or bronchiectasis in hospitalized patients with AECOPD. **(D)** Kaplan–Meier curve and log-rank test for day 30 outcomes among four phenotypes in hospitalized patients with AECOPD. Kaplan–Meier curve and log-rank test for day 30 outcomes (a) recurrent exacerbation (*P* = 0.5174) and (b) exacerbation-related readmission (*P* = 0.6853) among four phenotypes in hospitalized patients with AECOPD. ABCO, chronic obstructive pulmonary disease patients comorbid with asthma and bronchiectasis; ACO, asthma-chronic obstructive pulmonary disease overlap; AECOPD, acute exacerbation of chronic obstructive pulmonary disease; BCO, bronchiectasis-chronic obstructive pulmonary disease overlap; COPD, chronic obstructive pulmonary disease.

Kaplan–Meier curves and log-rank tests for recurrence of exacerbation and exacerbation-related readmission within 30 days after the index hospital discharge between ACO and BCO phenotype patients, between ACO or BCO phenotype patients and patients without asthma or bronchiectasis, and among the four phenotype patients are shown in Figure 3, respectively. Significant differences were not seen among these phenotypes.

## Discussion

In current manuscript, clinical characteristics and short-term prognosis profiles of hospitalized patients with COPD due to exacerbation comorbid with asthma and/or bronchiectasis, as well as those patients without the two comorbidities were fully analyzed. The prevalence of ACO, BCO, and ABCO phenotypes were 7.02, 10.22, and 1.31%, respectively.

Major findings of our analyses confirmed that patients with COPD comorbid with asthma and/or bronchiectasis or without showed distinct clinical features, particularly in age, gender, BMI, smoking status, prior allergic and COPD history, hospitalization due to exacerbation in the past year, comorbidities (e.g., pulmonary artery hypertension, lung cancer, failure of respiration, diabetes, gastroesophageal reflux disease, and anxiety or depression), levels of peripheral biomarkers (e.g., neutrophil, lymphocyte, eosinophil, creatinine, high-sensitivity C-reactive protein, fibrinogen, and N-terminal probrain natriuretic peptide), and lung function. Differences between ACO and BCO phenotypes were similar to previous research (16, 24, 27), but characteristics of ABCO phenotype were seldom described (23, 24). In our data, compared with ACO and BCO phenotype patients, the ABCO phenotype patients had significantly younger age, experienced more prior allergic episodes, and COPD diagnoses, but had a shorter COPD history, comorbid with more failure of respiration and anxiety or depression. Although demographic and clinical characteristics varied between the four phenotypes of patients, no statistical differences were observed with the short-term outcomes. The findings need to be validated using a larger and an independent sample as well as with longer follow-up information.

The prevalences of ACO and BCO phenotypes in our study were lower than previous reports (17–22, 36), maybe due to varied study populations, actual lower prevalence, different diagnosis criteria used, and underestimation of the diseases. CT scans for bronchiectasis and difficulties with diagnosing asthma clinically could also result in misclassification bias while the phenotypes were categorized. Additionally, differences in genetic backgrounds, allergens, chemical exposures, and dietary dissimilarities from other parts of the world may be alternative explanations, and if demography would play any role among Chinese patients living in other continents needs to be investigated. The latter assumptions should be tested *via* international multicenter collaboration between countries and ethnicities.

Furthermore, the entity of ACO phenotype has been debated for years, and the diagnosis criteria are developing. Cosio BG et al. used a composite criteria to define ACO, which included positive bronchodilator response, medical history of asthma, and higher levels of blood eosinophil and IgE (37). Hansen JE et al. suggested adopting a new bronchodilator response grading strategy based on FEV1 and FVC to identify the distinct ACO phenotype (38) and Fortis et al. (39) found that combined FEV1 and FVC bronchodilator response could be more sensitive to indicate an ACO phenotype, based on the observation that patients with asthma may have a greater bronchodilator response than those with COPD only. However, whether the bronchodilator response has diagnostic value in separating COPD and asthma is doubted (40). Validation studies are needed and consensus has to be achieved.

Current analyses were based on the largest ongoing multicenter registry on hospitalized patients with AECOPD in China. Characteristics and short-term prognoses of common but distinct phenotypes of COPD, i.e., ACO, BCO, ABCO phenotypes and those patients without the two diseases, were fully described. The findings of current analyses could provide the real-world evidence, as well as hints for disease management and further research. Meanwhile, several limitations of current analyses should be stated. First, some estimations of the associations lacked precision, i.e., a broad confidence interval might be due to the limited number of patients or events. Second, some potential impact factors and outcomes were not considered to avoid amplification of multiple testing and the type I error. Third, data on other common phenotypes such as chronic bronchitis and emphysema were not included. Additionally, some factors that would have influences on outcomes of interests were not collected, such as secondhand smoking, biomass fuel exposure, outdoor air pollution, etc, and alternative clinical outcomes including composite ones should be considered. With the ongoing recruitment and follow-up of the ACURE registry, a larger number of patients and long-term prognosis analyses in the future are possible.

## Conclusions

Current findings revealed that ACO, BCO, their overlaps (ABCO), and those patients without the two comorbidities had distinct clinical features, but did not differ in short-term prognoses. Further replication and validation in a larger and independent sample are warranted.

## Data Availability Statement

The raw data supporting the conclusions of this article will be made available from the corresponding authors on reasonable request, without undue reservation.

## Ethics Statement

The study involving human participants was reviewed and approved by the Institutional Review Board of the China-Japan Friendship Hospital (approval number: 2015-88). The participants provided their written informed consent to participate in this study.

## The China Acute Exacerbation of Chronic Obstructive Pulmonary Disease Inpatient Registry (Acure) Investigators

We deeply appreciated continuous supports and contributions from the following 161 hospitals and the local investigators: The First Affiliated Hospital of Hunan University of Medicine, Hunan Province; The First People's Hospital of Huaihua City, Hunan Province; Affiliated Hospital of Inner Mongolia University for the Nationalities, Inner Mongolia Autonomous Region; The People's Hospital of the Xishuangbanna Dai Nationality Autonomous Prefecture, Yunnan Province; Tongji Hospital, Tongji Medical College, Huazhong University of Science & Technology, Hubei Province; Zhangjiagang First People's Hospital, Jiangsu Province; The First Affiliated Hospital of Zhengzhou University, Henan Province; First Hospital of Shanxi Medical University, Shanxi Province; Central Hospital Affiliated to ShenYang Medical College, Liaoning Province; Liuzhou Workers Hospital, Guangxi Zhuang Autonomous Region; LongHua Hospital Shanghai University of Traditional Chinese Medicine, Shanghai City; The First Affiliated Hospital of Guangzhou University of Chinese Medicine, Guangdong Province; People's Hospital of Ji'an County, Jiangxi Province; Gongzhuling Central Hospital, Jilin Province; Chongqing Sixth People's Hospital, Chongqing City; Shanghai Putuo District Central Hospital (Putuo Hospital Affiliated to Shanghai University of Traditional Chinese Medicine), Shanghai City; The Second Affiliated Hospital of Tianjin University of Traditional Chinese Medicine, Tianjin City; Shanxi Fenyang Hospital, Shanxi Province; Taihe Hospital (Affiliated Taihe Hospital of Hubei University of Medicine), Hubei Province; Ulanqab Second Hospital, Inner Mongolia Autonomous Region; Nanjing Pukou District Central Hospital (Pukou Branch of Jiangsu Provincial People's Hospital), Jiangsu Province; Benxi Central Hospital, Liaoning Province; The Third People's Hospital of Jingdezhen, Jiangxi Province; Chongqing Jiulongpo District First People's Hospital, Chongqing City; Second Hospital of Shanxi Medical University, Shanxi Province; Ulanqab Central Hospital, Inner Mongolia Autonomous Region; Mianyang Central Hospital, Sichuan Province; Suining Central Hospital, Sichuan Province; The People's Hospital of Qitaihe, Heilongjiang Province; Affiliated Hospital of Zunyi Medical University, Guizhou Province; Inner Mongolia Baogang Hospital, Inner Mongolia Autonomous Region; Qiqihar Traditional Chinese Medicine Hospital, Heilongjiang Province; The Second Xiangya Hospital of Central South University, Hunan Province; Chengdu Qingbaijiang District People's Hospital, Sichuan Province; Minority Hospital of Guangxi Zhuang Autonomous Region (Minority Hospital Affiliated to Guangxi Medical University), Guangxi Zhuang Autonomous Region; Meihekou Central Hospital, Jilin Province; The People's Hospital of Yi County, Liaoning Province; The Central Hospital of Yongzhou, Hunan Province; Fujian Provincial Hospital, Fujian Province; The First Affiliated Hospital of Guiyang College of Traditional Chinese Medicine, Guizhou Province; The People's Hospital of Wanzhou District, Chongqing City; Renhe Hospital, Baoshan District, Shanghai City; The People's Hospital of Nanchuan, Chongqing City; Miyun District Hospital, Beijing City; The First People's Hospital of Chuzhou, Anhui Province; Shuguang Hospital Affiliated to Shanghai University of Traditional Chinese Medicine, Shanghai City; The Second Affiliated Hospital of Guangxi Medical University, Guangxi Zhuang Autonomous Region; Chengdu Second People's Hospital, Sichuan Province; 903 Hospital, Sichuan Province; The People's Hospital of Gaozhou, Guangdong Province; The Fourth Affiliated Hospital of Anhui Medical University, Anhui Province; Henan Provincial People's Hospital, Henan Province; The Second Affiliated Hospital of Shandong University of Traditional Chinese Medicine, Shandong Province; The Affiliated Hospital of Southwest Medical University, Sichuan Province; The Second People's Hospital of Guiyang, Guizhou Province; The Second Affiliated Hospital of Guilin Medical University, Guangxi Zhuang Autonomous Region; Inner Mongolia Xing'an League People's Hospital, Inner Mongolia Autonomous Region; Affiliated Hospital of Jiangxi University of Traditional Chinese Medicine, Jiangxi Province; The First Hospital of Kunming, Yunnan Province; The People's Hospital of Langfang City, Hebei Province; Shanghai Traditional Chinese Medicine-Integrated Hospital, Shanghai City; The First Hospital of Changsha, Hunan Province; The Fifth People's Hospital of Datong, Shanxi Province; Yichang Central People's Hospital, Hubei Province; Hebei Provincial Hospital of Traditional Chinese Medicine, Hebei Province; Fuling Central Hospital, Chongqing City; Jiangxi Pingxiang People's Hospital, Jiangxi Province; Shanxi Bethune Hospital (Shanxi Academy of Medical Sciences), Shanxi Province; Ths People's Hospital of Maoming, Guangdong Province; People's Hospital of Anshun City, Guizhou Province; China Resources WISCO General Hospital, Hubei Province; Qinghai Provincial People's Hospital, Qinghai Province; Shanghai Dongfang Hospital (Dongfang Hospital affiliated to Tongji University), Shanghai City; Xinjiang Uygur Autonomous Region People's Hospital, Xinjiang Uygur Autonomous Region; The People's Hospital of Cangnan County, Zhejiang Province; The Second Hospital of Harbin City, Heilongjiang Province; The Fourth Affiliated Hospital of Harbin Medical University, Heilongjiang Province; Shanghai Fifth People's Hospital, Fudan University, Shanghai City; Fujian Provincial People's Hospital, Fujian Province; Liuzhou City Liutie Central Hospital, Guangxi Zhuang Autonomous Region; XinSteel Center Hospital, Jiangxi Province; Liaocheng Hospital of Traditional Chinese Medicine, Shandong Province; Cangzhou People's Hospital, Hebei Province; The First Hospital of Lanzhou University, Gansu Province; Xinzhou People's Hospital, Shanxi Province; Affiliated Hospital of Guangdong Medical University, Guangdong Province; The Sixth Hospital of Beijing, Beijing City; Panjin Liaoyou Gem Flower Hospital (Liaohe Oilfield General Hospital), Liaoning Province; The People's Hospital of Nanping City, Fujian Province; The First People's Hospital of Qinzhou, Guangxi Zhuang Autonomous Region; The First Hospital of Tianjin, Tianjin City; Yichun People's Hospital, Jiangxi Province; The Central Hospital of Ningcheng County, Inner Mongolia Autonomous Region; The First Affiliated Hospital of Bengbu Medical College, Anhui Province; The Second People's Hospital of Lianyungang, Jiangsu Province; Gansu Gem Flower Hospital, Gansu Province; The People's Hospital of Lhasa, Tibet Autonomous Region; Anhui No. 2 Provincial People's Hospital, Anhui Province; Qingdao Municipal Hospital, Shandong Province; Maoming Hospital of Traditional Chinese Medicine, Guangdong Province; The People's Hospital of Pingliang, Gansu Province; Guizhou Provincial People's Hospital, Guizhou Province; Zhangjiajie City People's Hospital, Hunan Province; The Affiliated Hospital of Inner Mongolia Medical University, Inner Mongolia Autonomous Region; First Hospital of Qinhuangdao, Hebei Province; Chifeng Municipal Hospital, Inner Mongolia Autonomous Region; Jiading District Central Hospital, Shanghai City; The First Affiliated Hospital of Anhui University of Traditional Chinese Medicine, Anhui Province; The Affiliated Hospital of Shandong University of Traditional Chinese Medicine (Shandong Provincial Hospital of Traditional Chinese Medicine), Shandong Province; Yanbian No. 2 People's Hospital, Jinlin Province; The Third People's Hospital of Yichang City, Hubei Province; The Second Affiliated Hospital of Liaoning University of Traditional Chinese Medicine, Liaoning Province; Shenzhen Traditional Chinese Medicine Hospital, Guangdong Province; The First People's Hospital of Jiangxia District, Wuhan City, Hubei Province; The First People's Hospital of Xining, Qinghai Province; Xiamen Haicang Hospital, Fujian Province; Tibet Autonomous Region People's Hospital, Tibet Autonomous Region; The Third Hospital of Xiamen, Fujian Province; Nanjing Jiangning District Hospital of Traditional Chinese Medicine, Jiangsu Province; Sichuan Academy of Medical Sciences, Sichuan Provincial People's Hospital (East Hospital), Sichuan Province; Wuzhou Gongren Hospital, Guangxi Zhuang Autonomous Region; Wang Jing Hospital of China Academy of Chinese Medical Sciences, Beijing City; Shanghai Seventh People's Hospital, Shanghai City; The Fifth People's Hospital of Chongqing City, Chongqing City; The People's Hospital of Dongying City, Shandong Province; The Affiliated Hospital of Hangzhou Normal University, Zhejiang Province; The First Affiliated Hospital of Shantou University Medical College, Guangdong Province; Xinyu Hospital of Traditional Chinese Medicine, Jiangxi Province; Xishan Coal Electricity Group Gujiao Mining Area General Hospital, Shanxi Province; Ruikang Hospital Affiliated to Guangxi Universtiy of Chinese Medicine, Guangxi Zhuang Autonomous Region; The Third Affiliated Hospital of Guangzhou Medical University, Guangdong Province; Yu Tian Xian Zhong Yi Yuan, Hebei Province; People's Hospital of Changshou Chongqing, Chongqing City; Beijing Tiantan Hospital, Capital Medical University, Beijing City; General Hospital of Heilongjiang Provincial Agricultural Reclamation Bureau, Heilongjiang Province; The People's Hospital of Xishui County, Hubei Province; The First Hospital of Hunan University of Chinese Medicine, Hunan Province; The First People's Hospital of Huainan City, Anhui Province; The Second Affiliated Hospital of Nanjing Medical University, Jiangsu Province; Shanxi Provincial People's Hospital, Shanxi Province; West China Hospital of Sichuan University, Sichuan Province; Zhongshan Hospital Xiamen University, Fujian Province; Zheng Zhou Shi Zhong Yi Yuan, Henan Province; Xiangya Ping Mine Cooperative Hospital, Jiangxi Province; Northen Jiangsu People's Hospital, Jiangsu Province; Huzhou Hospital of Traditional Chinese Medicine affiliated to Zhejiang University of Traditional Chinese Medicine, Zhejiang Province; Guangdong Second Provincial General Hospital (Guangdong Provincial Emergency Hospital), Guangdong Province; Huainan Xinhua Hospital, Anhui Province; Third People's Hospital of Jiujiang City, Jiangxi Province; Panzhihua Hospital of Integrated Traditional Chinese and Western Medicine, Sichuan Province; The Central Hospital of Xuhui District, Shanghai City; Yueyang Integrated Traditional Chinese and Western Medicine Hospital Affiliated to Shanghai University of Traditional Chinese Medicine, Shanghai City; The Second Affiliated Hospital of Xiamen Medical College, Fujian Province; Hegang People's Hospital, Heilongjiang Province; Jiaozuo People's Hospital, Henan Province; Traditional Chinese Medicine Hospital of Kunshan, Jiangsu Province; The First People's Hospital of Nanning, Guangxi Zhuang Autonomous Region; Shandong Provincial Qianfoshan Hospital, Shandong Province; Beijing Hospital of Traditional Chinese Medicine, Capital Medical University, Beijing City; Tangshan People's Hospital, Hebei Province; Chongqing Traditional Chinese Medicine Hospital, Chongqing City.

## Author Contributions

JL conceptualized this study, did all statistical analyses, interpreted the data, wrote the first draft, and critically revised the manuscript. CL, KH, and SW took part in manuscript revision, project, and data management. TY and CW supervised the work, had full access to all of the data in the study, and took responsibility for the integrity of the work as a whole, from inception to the published article. All authors have read and approved the final manuscript to be published.

## Funding

This work was supported by the Chinese Academy of Medical Science (CAMS) Innovation Fund for Medical Sciences (CIFMS) (nos. 2021-I2M-1-049 and 2020-I2M-2-008) and the Chinese Academy of Medical Science (CAMS) Basic Scientific Research Business Fee Fund of Central Level Public Welfare Scientific Research Institutes (no. 2019TX320005). The funders had no role in study design, data collection and analysis, decision to publish, or preparation of the manuscript.

## Conflict of Interest

The authors declare that the research was conducted in the absence of any commercial or financial relationships that could be construed as a potential conflict of interest.

## Publisher's Note

All claims expressed in this article are solely those of the authors and do not necessarily represent those of their affiliated organizations, or those of the publisher, the editors and the reviewers. Any product that may be evaluated in this article, or claim that may be made by its manufacturer, is not guaranteed or endorsed by the publisher.

## Abbreviations

ABCO: chronic obstructive pulmonary disease patients comorbid with asthma and bronchiectasis
ACO: asthma-chronic obstructive pulmonary disease overlap
ACURE: the acute exacerbation of chronic obstructive pulmonary disease inpatient registry
AECOPD: acute exacerbation of chronic obstructive pulmonary disease
BCO: bronchiectasis- chronic obstructive pulmonary disease overlap
BMI: body mass index
CAT: chronic obstructive pulmonary disease assessment test
CI: confidence interval
COPD: chronic obstructive pulmonary disease
CT: computed tomography
DVT: deep venous thrombosis
FEV1: forced expiratory volume in 1 s
FVC: forced vital capacity
GOLD: the Global Initiative for Chronic Obstructive Lung Disease
HR: hazard ratio
ICU: intensive care unit
IQR: interquartile range
PE: pulmonary embolism
RICU: respiratory intensive care unit
SD: standard deviation
VTE: venous thromboembolism.
