# Supplementary material for: Comparison of Clinical Characteristics and Short-Term Prognoses Within Hospitalized Chronic Obstructive Pulmonary Disease Patients Comorbid With Asthma, Bronchiectasis, and Their Overlaps: Findings From the ACURE Registry
Source: Front Med (Lausanne). 2022 Feb 25;9:817048. doi: 10.3389/fmed.2022.817048 (PMC8914031; doi:10.3389/fmed.2022.817048)
Supplement: Supplementary file 1 [file Data_Sheet_1.docx]

Supplementary Material

**Supplementary file 1. Diagnoses of diseases and definitions of outcomes.**

**(1) Diagnoses of diseases**

**1) Spirometric COPD** was diagnosed with the presence of a post-bronchodilator forced expiratory volume in one second (FEV1) divided by forced vital capacity (FVC) with a value of less than 0.70 which indicated a persistent airflow limitation according to the Global Initiative for Chronic Obstructive Lung Disease (GOLD) 2021 Report (www.goldcopd.org).

Above standard diagnosis practice was applied across all participating centers.

**2) Asthma** was diagnosed with the presence of both variable expiratory airflow limitation and a characteristic pattern of respiratory symptoms for instance wheezing, shortness of breath (dyspnea), chest tightness and/or cough in adults according to the Global Initiative for Asthma (GINA) 2021 Report (www.ginasthma.org).

The variable expiratory airflow limitation included:

① Documented expiratory airflow limitation: at a time when FEV1 was reduced, confirmed that FEV1/FVC was reduced, i.e. usually > 0.75~0.80 in adults; and

② Documented excessive variability in lung function (one or more of the following):

- Positive bronchodilator reversibility test: increase in FEV1 of > 12% and > 200 mL (greater confidence if increase was > 15% and > 400 mL);
- Excessive variability in twice-daily peak expiratory flow (PEF) over two weeks: average daily diurnal PEF variability > 10%;
- Significant increase in lung function after four weeks of anti-inflammatory treatment: increase in FEV1 by > 12% and > 200 mL (or PEF by > 20%) from baseline after four weeks of treatment, outside respiratory infections;
- Positive exercise challenge test: fall in FEV1 of > 10% and > 200 mL from baseline;
- Positive bronchial challenge test: fall in FEV1 from baseline of ≥ 20% with standard doses of methacholine, or ≥ 15% with standardized hyperventilation, hypertonic saline or mannitol challenge;
- Excessive variation in lung function between visits: variation in FEV1 of > 12% and > 200 mL between visits, outside of respiratory infections.

Above standard diagnosis practice was applied across all participating centers.

**The ACO phenotype** was defined as spirometry-diagnosed COPD and asthma.

**3) Bronchiectasis** was diagnosed with the presence of both bronchial dilatation on computed tomography (CT) and clinical symptoms such as cough, sputum production and/or recurrent respiratory infection in adults according to the European Respiratory Society (Polverino E, et al. Eur Respir J. 2017) and British Thoracic Society guidelines (Pasteur MC, et al. Thorax. 2010).

The typical imaging parameters of bronchiectasis when high-resolution CT (HRCT) was used (Pasteur MC, et al. Thorax. 2010):

① Standard HRCT protocol, single detector CT scanner:

- Patient position: supine, breath holding at full inspiration; optional electrocardiograph gating 120-140 kV; 100-180 mAs (dependent on patient habitus); acquisition time < 1s;
- Beam collimation 1 mm; 1 cm intervals;
- Reconstruction with “very sharp” kernel.

② Volumetric HRCT protocol, 64-channel CT scanner:

- Patient position: supine, breath holding at full inspiration 120-140 kV; 120 effective mAs; rotation time 0.5 s;
- Detector collimation 0.6 mm; section thickness 1 mm; pitch 0.9;
- Reconstruction with “very or ultra sharp” kernel.

Above standard diagnosis practice was applied across all participating centers.

**The BCO phenotype** was confirmed by spirometric COPD and CT-based bronchiectasis.

**4) The ABCO phenotype** was termed as COPD both comorbid with asthma and bronchiectasis.

**(2) Definitions of outcomes**

**1) Length of index hospital stay (in days):** length of stay because of exacerbation of COPD during the index hospitalization.

**2) Recurrence of exacerbation within 30 days after the index hospital discharge (in events or rate):** outpatient or emergency department visit, or hospital readmission because of exacerbation of COPD within 30 days after the index hospital discharge.

**3) Exacerbation-related hospital readmission within 30 days after the index hospital discharge (in events or rate):** hospital readmission because of exacerbation of COPD within 30 days after the index hospital discharge.

**Supplementary file 2. Multivariable models adopted for stepwise selection of independent predictors.**

Multivariable linear regression model or Cox proportional hazards regression model was used to assess the associations of predictors with continuous outcome (i.e. length of hospital stay) or with the day 30 outcomes (i.e. recurrence of exacerbation and exacerbation-related hospital readmission) as appropriate. Variables that showed significant associations in univariable analyses (*P* value < 0.10), and factors (e.g. age, gender, BMI, smoking status, and FEV1) that previously had been reported to be associated with prognoses of COPD were further adjusted in the multivariable statistical models (Bellou V, et al. *BMJ (Clinical research ed)* 2019, 367: l5358). Stepwise selection scheme with an entry level of 0.10 and a stay level of 0.05 was applied. For individual regression model, variables with less than 10 events or with a missing rate lager than 30% were removed from the analysis.

**Candidate predicting factors included in the multivariable models for different phenotypes are as below:**

**(1) For the ACO phenotype:**

**1) Length of stay:** age (years), gender (male/female), BMI (kg/m^2^), smoking status (former/current/never), post-bronchodilator FEV1 (L), levels of neutrophil (*10^9^/L) and eosinophil (*10^9^/L).

**2) Recurrence of exacerbation:** age (years), gender (male/female), BMI (kg/m^2^), smoking status (former/current/never), post-bronchodilator FEV1 (L), community-acquired pneumonia (yes/no), osteoporosis (yes/no), cancer (yes/no), venous thromboembolism (yes/no) and level of eosinophil (*10^9^/L).

**3) Exacerbation-related readmission:** age (years), gender (male/female), BMI (kg/m^2^), smoking status (former/current/never), post-bronchodilator FEV1 (L), pulmonary interstitial fibrosis (yes/no), osteoporosis (yes/no), and emergency visit due to exacerbation in prior year (times).

**(2) For the BCO phenotype:**

**1) Length of stay:** age (years), gender (male/female), BMI (kg/m^2^), smoking status (former/current/never), post-bronchodilator FEV1 (L), education level (primary school or below/junior high school/senior high school/undergraduate or above), years of previous COPD diagnosis (years), peptic ulcer (yes/no), venous thromboembolism (yes/no), neutrophil (%), lymphocyte (%), PaCO2 (mmHg), and PaO2 (mmHg).

**2) Recurrence of exacerbation:** age (years), gender (male/female), BMI (kg/m^2^), smoking status (former/current/never), post-bronchodilator FEV1 (L), pulmonary heart disease (yes/no), gastroesophageal reflux disease (yes/no), hospitalization due to exacerbation in prior year (times), levels of red blood cell (*10^12^/L), direct bilirubin (umol/L) and alkaline phosphatase (U/L).

**3) Exacerbation-related readmission:** age (years), gender (male/female), BMI (kg/m^2^), smoking status (former/current/never), post-bronchodilator FEV1 (L), community-acquired pneumonia (yes/no), acute heart failure (yes/no), and hospitalization due to exacerbation in prior year (times).

**(3) For the ABCO phenotype:**

**1) Length of stay:** age over 80 years (yes/no), gender (male/female), BMI (kg/m^2^), smoking status (former/current/never), post-bronchodilator FEV1 (L), education level (primary school or below/junior high school/senior high school/undergraduate or above), cancer (yes/no), level of platelet (*10^9^/L), and PaCO2 (mmHg).

**2) Recurrence of exacerbation:** age (years), gender (male/female), BMI (kg/m^2^), smoking status (former/current/never), post-bronchodilator FEV1 (L), influenza or pneumonia vaccination within past five years (yes/no), years of previous COPD diagnosis (years), coronary heart disease (yes/no), anxiety or depression (yes/no), cerebrovascular disease (yes/no), levels of white blood cell (*10^9^/L), lymphocyte (*10^9^/L), hemoglobin (g/L), direct bilirubin (umol/L), and glucose (mmol/L).

**(4) For the COPD patients without asthma or bronchiectasis:**

**1) Length of stay:** age (years), gender (male/female), BMI (kg/m^2^), smoking status (former/current/never), post-bronchodilator FEV1 (L), education level (primary school or below/junior high school/senior high school/undergraduate or above), previous allergic episode (yes/no), influenza or pneumonia vaccination within past five years (yes/no), previous diagnosis of COPD, years of previous COPD diagnosis (years), pulmonary artery hypertension (yes/no), pulmonary interstitial fibrosis (yes/no), failure of respiration (yes/no), coronary heart disease (yes/no), chronic heart failure (yes/no), pulmonary heart disease (yes/no), acute heart failure (yes/no), cancer (yes/no), venous thromboembolism (yes/no), cerebrovascular disease (yes/no), ICU/RICU admission (yes/no), hospitalization due to exacerbation in prior year (times), emergency visit due to exacerbation in prior year (times), levels of white blood cell (*10^9^/L), lymphocyte (%), eosinophil (%), and alkaline phosphatase (U/L).

**2) Recurrence of exacerbation:** age (years), gender (male/female), BMI (kg/m^2^), smoking status (former/current/never), post-bronchodilator FEV1 (L), previous allergic episode (yes/no), failure of respiration (yes/no), chronic heart failure (yes/no), right bundle branch block (yes/no), pulmonary heart disease (yes/no), hospitalization due to exacerbation in prior year (times), emergency visit due to exacerbation in prior year (times), levels of white blood cell (*10^9^/L), neutrophil (%), lymphocyte (%), direct bilirubin (umol/L), alkaline phosphatase (U/L), gamma glutathione transpeptidase (U/L), and PaCO2 (mmHg).

**3) Exacerbation-related readmission:** age over 70 years (yes/no), gender (male/female), BMI (kg/m^2^), smoking status (former/current/never), post-bronchodilator FEV1 (L), previous diagnosis of COPD (yes/no), community-acquired pneumonia (yes/no), failure of respiration (yes/no), right bundle branch block (yes/no), pulmonary heart disease (yes/no), diabetes (yes/no), hospitalization due to exacerbation in prior year (times), emergency visit due to exacerbation in prior year (times), levels of white blood cell (*10^9^/L), platelet (*10^9^/L), neutrophil (%), lymphocyte (%), direct bilirubin (umol/L), and PaCO2 (mmHg).
